# An Unexpected Recurrent Transmission of Rift Valley Fever Virus in Cattle in a Temperate and Mountainous Area of Madagascar

**DOI:** 10.1371/journal.pntd.0001423

**Published:** 2011-12-20

**Authors:** Veronique Chevalier, Toky Rakotondrafara, Marion Jourdan, Jean Michel Heraud, Harena Rasamoelina Andriamanivo, Benoit Durand, Julie Ravaomanana, Pierre E. Rollin, René Rakotondravao

**Affiliations:** 1 CIRAD, International Centre of Research in Agronomy for Development (AGIRs Unit), Montpellier, France; 2 FOFIFA-DRZV, BP 04, Antananarivo, Madagascar; 3 Virology Unit, Institut Pasteur de Madagascar, BP 1274, Antananarivo, Madagascar; 4 Agence Nationale de Sécurité Sanitaire (ANSES), Laboratoire de Santé Animale, Maisons-Alfort, France; 5 Viral Special Pathogens Branch, Division of High Consequence Pathogens and Pathology, Center for Disease Control and Prevention, Atlanta, Georgia, United States of America; USAMRIID, United States of America

## Abstract

Rift Valley fever is an acute, zoonotic viral disease of domestic ruminants, caused by a phlebovirus (Bunyaviridae family). A large outbreak occurred in Madagascar in 2008–2009. The goal of the present study was to evaluate the point prevalence of antibodies against Rift Valley Fever Virus (RVFV) in cattle in the Anjozorobe district, located in the wet and temperate highland region of Madagascar and yet heavily affected by the disease, and analyse environmental and trade factors potentially linked to RVFV transmission. A serological study was performed in 2009 in 894 bovines. For each bovine, the following variables were recorded: age, location of the night pen, minimum distance from the pen to the nearest water point and the forest, nearest water point type, and herd replacement practices. The serological data were analyzed using a generalized linear mixed model. The overall anti-RVFV IgG seroprevalence rate was 28% [CI95% 25–31]. Age was statistically linked to prevalence (p = 10^−4^), being consistent with a recurrent RVFV circulation. Distance from the night pen to the nearest water point was a protective factor (p = 5.10^−3^), which would be compatible with a substantial part of the virus transmission being carried out by nocturnal mosquito vectors. However, water point type did not influence the risk of infection: several mosquito species are probably involved. Cattle belonging to owners who purchase animals to renew the herd were significantly more likely to have seroconverted than others (p = 0.04): cattle trade may contribute to the introduction of the virus in this area. The minimum distance of the night pen to the forest was not linked to the prevalence. This is the first evidence of a recurrent transmission of RVFV in such an ecosystem that associates a wet, temperate climate, high altitude, paddy fields, and vicinity to a dense rain forest. Persistence mechanisms need to be further investigated.

## Author Summary

Rift Valley fever (RVF) is a viral disease of domestic ruminants, which may affect humans. The RVF virus (RVFV) may be transmitted either by mosquitoes or through direct contact with vireamic body fluids or products. Until now, this disease had been described in arid, hot and irrigated or tropical areas. Performed in the year following the 2008–2009 RVFV outbreak in Madagascar, this study demonstrates for the first time a regular and intense transmission of this disease in a temperate and mountainous region.The area chosen as a pilot project shows that cattle are regularly and heavily affected in the highlands of Madagascar. Statistical analyses suggest that (i) a substantial part of the transmission is due to mosquito vectors; (ii) many mosquito species such as *Culex* and *Anopheles*, are probably involved in the transmission; (iii) cattle trade, by a regular introduction of the virus via herds coming from infected areas of the island, may explain the recurrence of the disease in this region. Further investigations are needed to understand the mechanisms of transmission of the disease, and design and implement appropriate surveillance and control measures in this area.

## Introduction

Rift Valley fever (RVF) is an acute, zoonotic viral disease, caused by a phlebovirus belonging to the Bunyaviridae family [1]. It mainly affects domestic ruminants such as sheep, goats and cattle [2]. The main clinical signs of the disease are high mortality rates, especially in new-born sheep and goats, and abortion in pregnant animals. Humans can develop RVF after exposure to tissues, blood or body fluids or through the bite of an infected mosquito. Infection in humans is usually associated with moderate influenza-like illness, but severe complications occur in a small proportion of patients in which the death rate may be high. RVF virus (RVFV) is transmitted from ruminant to ruminant either by mosquitoes – the main mosquito vectors being from the *Aedes* and *Culex* genera [3], or theoretically, but never demonstrated, through direct contact. The respective contribution of both transmission routes remain unevaluated and probably vary from one ecological context to another. Veterinary inactivated and attenuated vaccines exist, but they are not widely used, and there is no treatment, either for humans or domestic animals.

The disease is endemic in numerous African countries. A large epidemic occurred in 2006–2007 in the Horn of Africa, first in Kenya [4], Tanzania and Somalia [5], then in Sudan [6]. The last outbreaks occurred in Madagascar in 2008 [7] and South Africa in 2010 [8]. Sporadic animal cases were also reported in Botswana [9] and Namibia [10].

The epidemiology of RVF is complex and only partially understood. The disease was reported in three epidemiological systems: (i) dambo areas (East, and some parts of Southern Africa where there are referred to as pans), (ii) semi-arid areas, and (iii) irrigated areas. Dambos are shallow depressions, often located near rivers, which fill with water during the rainy season. In these regions, a correlation between heavy rainfall and RVF outbreaks was clearly demonstrated [11]. Transmission from one mosquito generation to another, namely “vertical transmission” has been demonstrated with *Aedes (Neomelaniconion) mcintoshi* [12]. In addition, the virus may survive in desiccated eggs during inter-epizootic and/or dry/cold periods. Thanks to these two mechanisms, and to extreme rainy events, the disease may re-emerge every 5 to 15 years with only few seroconversion signs or reported clinical cases during the inter-epizootic period [13], [14]. The semi-arid areas in which the disease has been reported are characterized by temporary water points, such as found in northern Senegal or Mauritania. In these areas, the virus persistence mechanisms remain unclear. They could be related to the survival of the virus in *Aedes* mosquitoes, as demonstrated in East Africa, or to the regular introduction of the virus by nomadic herds coming from neighbouring endemic areas. Neither possibility is mutually exclusive [15]. The irrigated areas concerned include the Nile delta (Egypt) and the Senegal River valley (Senegal, Mauritania), where permanent water bodies favor the development of *Culex* populations, and thus year –long viral transmission [16]. In some ecosystems such as South Africa or Zimbabwe [17], [18], virus circulation also could be maintained between mosquito vectors and wild small or large mammals in sylvatic cycles.

In Madagascar, RVFV was isolated for the first time in 1979 from pools of mosquitoes captured during the rainy season in the primary rain forest of Perinet, Moramanga district [19]. Human and animal RVF outbreaks occurred during the rainy season in Vavatenina and Fenoarivo Antsinanana districts in March 1990 and around Antananarivo, the capital, from February to April 1991 [20]. Antigenic and molecular analysis of isolates showed that RVFV strains obtained in 1979 were closely related to both Egyptian 1979 isolates and Zimbabwean 1974 isolates, while those isolated in 1991 were closer to eastern/central African strains [20]–[22].

The first human RVF case of the 2008 outbreak was reported in January in Tolagnaro city, southern Madagascar, from specimen collected at the sentinel surveillance site. By 15 June 2008, the Ministry of Health of Madagascar had reported 417 suspected RVF cases, 59 laboratory confirmed human cases, and 19 deaths suspected to be due to RVF infection [7]. Most regions of the island were infected. Anjozorobe district was heavily affected by the outbreak (Reynes JM, personal communication). This region, located 80 km north of Antananarivo, is part of the wet and temperate highland region of Madagascar. It is composed of a dense, evergreen forest and an agricultural zone, where there are rice fields favorable to *Culex* mosquito populations and livestock farming is widespread.

Due to a rather cool weather during winter, a continuous transmission of the virus between mosquitoes and cattle is unlikely. As suspected in other parts of the world (e.g., bats in Guinea [23] and rodents in Senegal and South Africa [17], [24], [25]), the existence of a wild reservoir located in the forest may explain the persistence of the virus during inter-epizootic periods and the recurrence of the disease in this region. Alternatively, livestock trade may be involved in this resurgence given that the Anjozorobe region is connected to most of the important breeding areas in the country.

The goal of the present study was to evaluate the point prevalence rate of antibodies against RVFV in cattle in the Anjozorobe district and identify environmental and trade factors potentially linked to RVFV transmission to infer epidemiological implications of RVFV circulation and persistence in this area.

## Methods

### The study area

**Figure 1:**
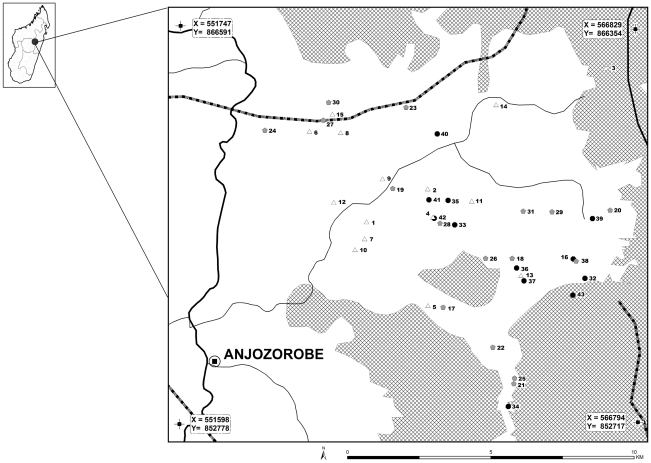
Location of villages included in the serological survey and corresponding IgG seroprevalence rates. Thick line is the main road. Thin lines are secondary roads. Dashed lines are water systems. Moist forests are represented by gridded areas. Empty triangles represent villages with an IgG seroprevalence rate ranging from 0 to 22,2% (n = 15); grey diamonds represent villages with an IgG seroprevalence rate ranging from 22,21 to 33,3% (n = 16); black circles represent villages with an IgG seroprevalence rate ranging from 33,4 to 71,4% (n = 12). Village identification numbers (n = 43) are provided in Online supporting information file, Table S1. Source: @FTM BD 2000, CIRAD.

The study area is located within the Anjozorobe district, 80 km north of Antananarivo, the capital of Madagascar Island (defined by the geographical coordinates of the Figure 1). This region holds some of the last large and unfragmented remnants of the central highland's natural ecosystems, including highland forests, watersheds and lakes (Figure 1). The study area, mainly composed of wetlands, rice fields, and crop fields lies beside the Anjozorobe forest corridor which is one of the last vestiges of dense rain forest in Madagascar. Two types of forest are present: high altitude humid forest (over 1,500 m) and mountain humid forest (800–1,500 m). The climate is temperate and wet, characterized by a cold season with frequent but slight rainfall, from April to September, and a warm, wet season with high rainfall, from October to April. The average annual temperature is 18°C. November is the warmest month, with an average maximum temperature of 27°C, and August the coldest with an average minimum temperature of 9°C [26]. The forest corridor is on the continental divide line between the east and west side of Madagascar Island. The region is crossed by many water systems that constitute an important water resource irrigating the surrounding regions [26].

In this area, livestock farming, largely of cattle, is crucial for the local population's subsistence, contributing directly and indirectly to food security and nutrition. Livestock provide products (milk and meat) for consumption, and is a source of valuable goods and services, e.g. transport, manure for fertilizing, ploughing, rice stamping and income from trade of products. Cattle are also slaughtered regularly for religious feasts and are used as a form of savings.

### Sampling and definition of risk factors

In collaboration with the Malagasy Veterinary Services, an exhaustive census of cattle herds was performed in the study area in May 2009. Meetings were organized with farmers to explain the goals of the study and the decision to participate was taken at the village level. Informed consent was given orally and documented in questionnaires. For cultural reasons, written consent could not be obtained. The size of the cattle population in this area was estimated by the Veterinary Services to be 2,000 animals. Thus, considering a design effect of 2 that takes into account the village clustering, and to estimate a point prevalence of 20% with a 95% level of confidence, we needed to sample at least 1,020 animals (exact error 3%). In each participating village, blood samples were collected from randomly chosen cattle. Each participating owner was the source of data collected in a standardized questionnaire. The age of each sampled animal was recorded. The potential role of 3 environmental risk factors was investigated: the minimum distance from the night pen to the nearest water point, the type of the nearest water point, and the minimum distance from the night pen to the nearest forest boundary. The exact locations of the night pens and the distances from these night pens to the nearest water point and nearest forest boundary were recorded and calculated using a Global Positioning System (GPS) device. With regards to the type of water point, 3 modalities were recorded, namely “pond”, “rice field” and “river”. In addition to the 3 above–mentioned parameters, owners were questioned about their herd replacement practices. The binary variable -auto-renewal *vs* purchase- was recorded. When purchased, the exact origin of the cattle could not be obtained. However most of them were bought in the Anjozorobe area (H. Rasamoelina, pers.com).

In the field, blood samples were centrifuged after collection, then transported to the Institut Pasteur in Madagascar (Antananarivo) and stored at −20°C until they were analysed.

### Serological analysis

Samples were tested for anti-RVFV immunoglobulin (Ig) M and IgG using a previously described ELISA test [7], [27]. The ELISA assays were completed by using inactivated RVFV-infected vero E6 cell antigens and uninfected Vero E6 cell antigens. Each serum was 4-fold diluted, namely 1∶100, 1∶400, 1∶1600 and 1∶6400. Samples were considered positive only if the adjusted sum of optical densities, defined for each dilution as the difference between the cumulative sum of optical densities minus the background absorbance of uninfected control Vero E6 cells, and the titers were above pre-established conservative cut-off values, respectively ≥0.75 and ≥400 for IgM and ≥0.95 and ≥400 for IgG.

### Statistical analysis

The serological data were analyzed using a generalized linear mixed model (glmmML library, R software), where the individual serological status was the binomial response, and the previously mentioned variables (age, minimum distance from the night pen to the nearest water point type, minimum distance from the night pen to the nearest forest boundary, type of the nearest water point, and herd replacement practices) were the explicative factors. The breeder was included as a random effect to take into account the clustering of sampled animals. The significance of the cluster effect was tested using a bootstrap approach (n = 2000) [28].

## Results

**Figure 2:**
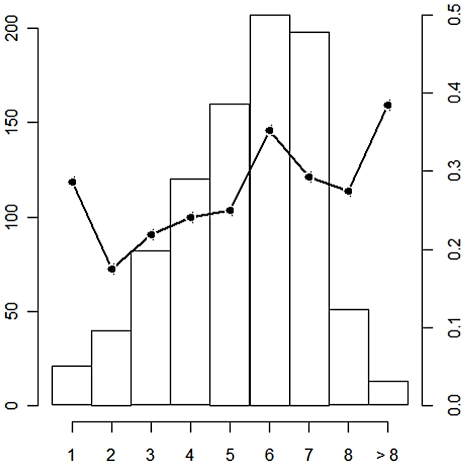
Number of sample animals and IgG seroprevalence rate according to age. The number of sampled animals is given by the histogram, and the black line represents the IgG prevalence rate. Thirteen animals older than 8 were aggregated in the class >8. Age of animals is given by the x axis. The left y axis represents the number of sampled animals for each age class. The right y axis provides the IgG prevalence rate for each age class.

Due to field constraints, the provisional sampling could not be achieved: a total of 894 bovines were sampled between early May and mid-June 2009, belonging to 258 breeders coming from 43 villages of 51 villages existing in the area (See online information file, Table S1). Cattle ages ranged from 1 to 18 years, with the majority of sampled animals less than 8 years old (Figure 2). Overall anti-RVFV IgG seroprevalence rate was 28% [IC95% 25–31] (See online information file, Table S1). This rate varied among villages, from 0 to 71.4% (See online information file, Table S1). The overall anti-RVFV IgM seroprevalence rate was 0.8% [IC95% 0.0–1.0]. The 7 anti-RVFV IgM positive animals were distributed among herds from 6 villages and 5 of the animals belonged to breeders that do not purchase new animals to renew their herd.

The IgG seropositivity rate of animals was statistically linked to age (p = 0.0001) with an OR of 3.4 for an age difference of 5 years suggesting a recurrent and intense RVFV circulation in this area (Table 1). The minimum distance to the nearest water point ranged from 10 to 3,000 meters. This distance was a protective factor (p = 0.005) since prevalence rate was negatively correlated to the distance. The odds of seropositivity were divided approximately by 10 when the distance to the nearest water point was increased by 500 meters (OR: 0.08, Table 1). Five hundred thirty three bovines belonged to breeders that did not purchase any animal. The anti-RVFV IgG seroprevalence within this population was 27.8%. The anti-RVFV IgG seroprevalence of bovines belonging to breeders that purchase to renew their herd was 28.1%. In the multivariate model, belonging to owners who buy animals to renew (at least partially) their herd was a risk factor (p = 0.04). The minimum distance of the night pen to the forest ranged from 100 to 10,000 meters. This latter parameter as well as the water point type had no effect on the prevalence rate. The breeder random effect was not significant (p = 0.06).

**Table 1:** Results of the generalized linear mixed-effect model, with breeder as a random effect.

Variable	Modalities	OR (95%CI)	p-value
Age		3.4^a^ (1.8–6.4)	10^−4^
Minimum distance to water point		0.08^b^ (0.01–0.48)	5.10^−3^
Water point type^c^			
	River	NS^d^	NS
	Paddy field	NS	NS
Minimum distance to the forest		NS	NS
Replacement practices		33 (1–992)	0.04

a Change of the odds of seropositivity when the age of animals is increased by 5 years.

b Change of the odds of seropositivity when the distance to the nearest water point is increased by 500 m.

c Pond as the reference.

d Non Significant.

## Discussion

The overall observed anti-RVFV IgG prevalence was concordant with a serosurvey performed after the 1991 outbreak in Mangamila, located 20 kms south of Anjozorobe [29]. Similarly, a national serosurvey was performed after the 2008 outbreak: the seroprevalence rate was estimated to be 25.8% and 24.7% respectively in cattle (n = 3437) and small domestic ruminants (n = 989) [30].

Only 7 bovines were found positive for IgM against RVFV. In the majority of cases, anti-RVFV IgM antibodies do not persist beyond the 50th day after infection [31], [32]. The sampling of the anti-RVFV IgM positive bovines was performed between early May and mid-June 2009. This suggests that these infections most probably occurred between early February 2009, and mid March, corresponding to the middle of the wet, warm season (October to April) and the apparent end of the second transmission wave. A high herd immunity level induced by the first outbreak in 2008 associated with an early elimination of IgM might explain this small number of new infections. Five of these 7 anti-RVFV IgM positive bovines belonged to breeders that do not purchase to renew their herds: these 5 infections were caused by local viral activity.

The first potential risk factor was the minimum distance to the nearest water point. The main known RVFV vectors are from *Culex* and *Aedes* genera whose main activity period is crepuscular and nocturnal [33]: given that cattle come back from field in late afternoon and spend the night in pens, we assume that the pens are where infection by mosquito bites occurs. The second potential risk factor was the minimum distance to the forest. The existence of a sylvatic cycle between mosquitoes and wild reservoirs living in the forest could explain the persistence and the re-emergence of the virus in the area. In that case, bovines living close to the forest would be more exposed to infectious mosquito bites than others. The water point type was identified as a third potential risk factor. Indeed, the ecology of *Culex* and *Aedes mosquito* vectors is rather different and closely related to the water point filling and drying rhythm. *Aedes* females lay their eggs on the muddy banks of ponds. These eggs may survive several years in desiccated mud. When flooded again during pond filling, there is a massive hatch of eggs. In contrast, *Culex* eggs cannot survive to desiccation, and need to be permanently in water to develop and hatch [33].

An increased distance to the nearest water point appears to be a protecting factor, suggesting that a substantial part of the virus transmission could be carried out by mosquito vectors. As a matter of fact, 6 mosquito genera, namely *Aedes*, *Anopheles*, *Coquillettidia*, *Culex*, *Eretmapodites and Mansonia* have been proven to be capable of RVFV infection and transmission in the laboratory and/or were found infected in the wild [34]–[36]. These genera are all present in Madagascar [19], [37]. Among them, unfed females of 3 species have been found infected by RVFV in another part of the highlands, the Fianarantsoa region, located 400 km far from the Anjozorobe area: *An. coustani*, *An. squamosus*, and *Cx. antennatus* [38]. *An. coustani* and *An. squamosus* were found infected in Madagascar in 1979 [37] and *Cx. antennatus* in Kenya from 1981 to 1984 [12]. However vector competence was experimentally demonstrated only for *Cx. antennatus* in Egypt [39]. According to our results, the type of water point has no effect on the prevalence rate. This corroborates the assumption that several mosquito species with different biology and ecology may be involved in the transmission cycle in this area: paddy fields are favourable to *An. coustani* and *Cx. antennatus* [40] whereas water-filled hoofprints in the stream bank favour the development of *An. squamosus* [41]. Further entomological studies are needed to confirm this assumption and the involvement of these mosquito species in the RVF epidemiological cycle in Madagascar highlands.

The potential role of small mammals as a RVFV reservoir, especially rodents, has been suspected but never demonstrated. A high mortality rate among rodents (*Arvicanthis abyssinicus nairobae* and *Rattus rattus kijabius*) was observed on farms affected during the 1930 epizootic in Kenya [2]. The existence of viraemia was demonstrated in *Arvicanthis abyssinicus* after inoculation with RVFV [42]. In Egypt, RVFV was detected by RT-PCR in the blood of 29 *Rattus rattus* individuals among 300 sampled [43]. Serological evidence of RVFV infection also was observed in *Mastomys sp.*, *Arvicanthis niloticus* and *Aethomys namaquensis* in Senegal [24]. In South Africa, serological and experimental studies showed that *Aethomys namaquensis* can act as an amplifying host for RVFV during inter-epizootic periods [17]. Given high biodiversity of rodents and small mammals in the Anjozorobe forest corridor, including an important *Rattus rattus* population [26], the persistence and the resurgence in 2008 of the RVFV in this particular ecosystem could be explained by the existence of a wild reservoir and a sylvatic cycle involving vectors that preferentially feed on rodents and occasionally feed on cattle, thereby acting as bridge vectors. However, our results clearly show that livestock living close to the forest or even grazing in the forest have the same level of exposure as livestock that do not live close to or graze in the forest. To date no molecular or serological signature was detected on rodents or small mammals of the area, but ongoing research is examining the hypothesis.

Ruminant trade has often been associated with local, regional, even continental dissemination of RVFV, as described from Sudan to Egypt during the 1970s [44], and from the Horn of Africa to the Arabian Peninsula in 2000 [45]. Regarding the Anjozorobe area, bovines belonging to owners who replace their herds by purchasing animals were significantly more antibody-positive than others: animal introduction in a herd is a risk factor and the virus may, conceivably, be introduced by a viraemic animal, coming from either another village in the study area or a more distant location. In both cases, the transmission to the cattle in the herd may occur either by a direct or vectorial route. Due to the lack of data on bovine origins, the extent to which these introductions participate in the maintenance of the RVF cycle in the Anjozorobe area remains unknown. The spatial clustering of sampled bovines was taken into account including a breeder random effect in the model. Indeed we considered that the level of exposition to infection of bovines parked in the same night pen was correlated, whether this infection occurs by mosquito bites or direct contact. Statistical analyses showed that this effect was not significant.

This is the first serological assessment of RVFV transmission mechanisms in such an ecological context, which associates a wet, temperate climate, high altitude with mountainous reliefs, paddy fields, and vicinity to a dense rain forest. The epidemiological cycle probably involves several vectors – at least from *Aedes*, *Culex*, and *Anopheles* genera - and a combination of different mechanisms for virus persistence and diffusion, their respective importance varying with the season, the prevalence rate of the cattle, and the dates of religious festivals that increase the cattle trade flow. As shown in previous studies [7], [30], the disease circulates in different ecozones of the country, including a Sahel-like ecosystem in the south, to the very moist lowlands of the eastern coast, the moist lowland of the northwest region and the temperate climate of the central highlands. Jeanmaire et al. (2011) suggested that RVFV circulation was endemic in both the southern and north-western areas, and that these two areas may act as a virus source for the rest of the country [30]. The large distribution of the disease would then be explained by a large diffusion of the virus through the trade of bovines which is known to be extensive and sometimes uncontrolled in the island. Our results strongly suggest that RVFV circulation is also recurrent in the Anjozorobe area, suggesting a third RVF epidemiological system. Each epidemiological system may be linked to the others by the animal trade network. A global and yearlong circulation on the whole country could then be maintained by this network, itself possibly linked with other East-African countries, as suggested by recent molecular epidemiology results [46]. The reason why the disease re-emerged in 2008 in this area remains unknown and the risk of a new outbreak there has not been evaluated. Further studies should be carried out to describe and model this cycle in order to implement relevant surveillance, risk prediction and control measures.

## Supporting Information

Table S1 **Sample size, replacement practices ratio and observed serological IgM and IgG prevalence of Rift Valley fever on cattle, Anjozorobe district, Madagascar.**
(DOC)

## References

[1] Xu F, Liu D, Nunes M, Da Rosa A, Tesh R (2007). Antigenic and genetic relationships among Rift Valley fever virus and other selected members of the genus Phlebovirus (Bunyaviridae).. Am J Trop Med Hyg.

[2] Daubney R, Hudson J, Garnham P (1931). Enzootic hepatitis or Rift Valley fever: an undescribed disease of sheep, cattle and man from East Africa.. Journal of Pathology and Bacteriology.

[3] Wilson ML (1994). Rift Valley fever virus ecology and the epidemiology of disease emergence.. Ann NY Acad Sci.

[4] CDC (2007). Rift Valley fever outbreak—Kenya, November 2006–January 2007.. MMWR.

[5] WHO (2007). Outbreaks of Rift Valley fever in Kenya, Somalia, and United Republic of Tanzania, December 2006–April 2007.. Weekly epidemiological record.

[6] Adam A, Karsany M, Adam I (2010). Manifestations of severe Rift Valley fever in Sudan.. Int J Infect Dis.

[7] Andriamandimby S, Randrianarivo-Solofoniaina A, Jeanmaire E, Ravolomanana L, Razafimanantsoa L (2010). Rift Valley fever during rainy seasons, Madagascar, 2008 and 2009.. Emerg Inf Dis.

[8] OIE-WAHID (2010). Rift Valley fever in South Africa.. http://www.oie.int/wahis/public.php?page=single_report&pop=1&reported=9491.

[9] OIE-WAHID (2010). Rift Valley fever, Bostwana.. http://www.oie.int/wahis/public.php?page=singlereportpop=1.

[10] OIE-WAHID (2010). Rift Valley fever, Namibia.. http://web.oie.int/wahis/public.php?page=single_report&pop=1&reported=925811.

[11] Linthicum K, Anyamba A, Tucker C, Kelley P, Myers M (1999). Climate and satellite indicators to forecast Rift Valley fever epidemics in Kenya.. Science.

[12] Linthicum KJ, Davies FG, Kairo A, Bailey CL (1985). Rift Valley fever virus (family *Bunyaviridae*, genus *Phlebovirus*). Isolations from *Diptera* collected during an inter-epizootic period in Kenya.. J Hyg.

[13] Martin V, De Simone L, Lubroth J, Ceccato P, Chevalier V (2007). Perspectives on using remotely sensed imagery in predictive veterinary epidemiology and Global Early Warning Systems.. Geospatial Health.

[14] Rostal MK, Evans AL, Sang R, Gikundi S, Wakhule L (2010). Identification of potential vectors of and detection of antibodies against Rift Valley fever virus in livestock during interepizootic periods.. Am J Vet Res.

[15] Chevalier V, Lancelot R, Thiongane Y, Sall B, Mondet B (2005). Incidence of Rift Valley fever in small ruminants in the Ferlo pastoral system (Senegal) during the 2003 rainy season.. Emerg Inf Dis.

[16] Meegan JM, Hoogstraal H, Moussa MI (1979). An epizootic of Rift Valley fever in Egypt in 1977.. Vet Rec.

[17] Pretorius A, Oelofsen MJ, Smith MS, Van der Ryst E (1997). Rift Valley fever virus: a seroepidemiologic study of small terrestrial vertebrates in South Africa.. Am J Trop Med Hyg.

[18] Anderson EC, Rowe LW (1998). The prevalence of antibody to the viruses of Bovine Virus Diarrhoea, Bovine Herpes Virus 1, Rift Valley Fever, Ephemeral Fever and Bluetongue and to *Leptospira sp* in free-ranging wildlife in Zimbabwe.. Epidemiol Infect.

[19] Fontenille D, Rodhain F, Digoutte JP, Mathiot C, Morvan J (1989). Transmission cycles of the West Nile virus in Madagascar, Indian Ocean.. Ann Soc Belg Med Trop.

[20] Morvan J, Rollin PE, Laventure S, Rakotoarivony I, Roux J (1992). Rift Valley fever epizootic in the central highlands of Madagascar.. Res Virol.

[21] Sall AA, Zanotto P, Vialat P, Sene O, Bouloy M (1998). Origin of 1997–98 Rift Valley fever outbreak in East Africa.. Lancet.

[22] Bird BH, Khristova ML, Rollin PE, Ksiazek TG, Nichol ST (2007). Complete genome analysis of 33 ecologically and biologically diverse Rift Valley fever virus strains reveals widespread virus movement and low genetic diversity due to recent common ancestry.. J Virol.

[23] Boiro I, Konstaninov OK, Numerov AD (1987). Isolation of Rift Valley fever virus from bats in the Republic of Guinea.. Bull Soc Pathol Exot Filiales.

[24] Diop G, Thiongane Y, Thonnon J, Fontenille D, Diallo M (2000). The potential role of rodents in the enzootic cycle of Rift Valley fever virus in Senegal.. Microbes and Infection.

[25] Evans A, Gakuya F, Paweska JT, Rostal M, Akoolo L (2007). Prevalence of antibodies against Rift Valley fever virus in Kenyan wildlife.. Epid Infect.

[26] Goodman SM, Raselimanana AP, Wilmé L (2007). Inventaires de la faune et de la flore du couloir forestier d'Anjozorobe-Angavo..

[27] Madani TA, Al-Mazrou Y, Al-Jeffri H, Mishkas A, Al-Rabeah A (2003). Rift Valley fever epidemic in Saudi Arabia: epidemiological, clinical and laboratory characteristics.. Clin Infect Dis.

[28] Sinha SK (2009). Bootstrap test for variance components in generalized linear mixed model.. The Canadian Journal of Statistics.

[29] Morvan J, Rollin PE, Roux J (1992). Situation de la fièvre de la Vallée du Rift à Madagascar en 1991. Enquêtes séro-épidémiologiques chez les bovins.. Rev Elev Med Vet Pays Trop.

[30] Jeanmaire EM, Rabenarivahiny R, Biarmann M, Rabibisoa L, Ravaomanana F, Randriamparany T (2011). Prevalence of Rift Valley fever infection in ruminants in Madagascar after the 2008 outbreak.. Vector Borne Zoonotic Dis.

[31] Morvan J, Rollin PE, Laventure S, Roux J (1992). Duration of immunoglobulin M antibodies against Rift Valley fever virus in cattle after natural infection.. Trans R Soc Trop Med Hyg.

[32] Paweska J, Burt F, Anthony F, Smith S, Grobbelaar A (2003). IgG-sandwich and IgM-capture enzyme-linked immunosorbent assay for detection of antibody to Rift Valley fever in domestic ruminants.. J Virol methods.

[33] Beaty BJ, Marquardt WC (1996). The biology of disease vectors.

[34] Pépin M, Bouloy M, Bird BH, Kemp A, Paweska J (2010). Rift Valley fever (Bunyaviridae: Phlebovirus): an update on pathogenesis, molecular epidemiology, vectors, diagnostics and prevention.. Vet Res.

[35] Logan TM, Linthicum KJ, Thande PC, Wagateh JN, Roberts CR (1991). Mosquito species collected from a marsh in western Kenya during the long rains.. J Am Mosq Control Assoc.

[36] Meegan JM, Bailey CH, Monath TP (1988). Rift Valley fever.. The Arboviruses: epidemiology and ecology, vol. 4.

[37] Clerc Y, Rodhain F, Digoutte J, Albignac R, Coulanges P (1982). Le programme exploratoire arbovirus de l'Institut Pasteur de Madagascar.. Arch Inst Pasteur de Madagascar.

[38] Ratovonjato J, Olive MM, Tantely LM, Andrianaivolambo L, Tata E (2010). Detection, Isolation, and Genetic Characterization of Rift Valley Fever Virus from *Anopheles* (Anopheles) *coustani*, *Anopheles* (Anopheles) *squamosus*, and *Culex* (Culex) *antennatus* of the Haute Matsiatra Region, Madagascar.. Vector Borne Zoonotic Dis.

[39] Turell MJ, Presley S, Gad A, Cope S, Dohm D (1996). Vector competence of Egyptian mosquitoes for Rift Valley fever virus.. Am J Trop Med Hyg.

[40] Muturi E, Shililu J, Jacob B, Mwangangi J, Mbogo C (2008). Diversity of rice land mosquitoes and factors Affecting their occurrence and distribution in Mwea, Kenya.. J Am Mosq Control Assoc.

[41] Shililu J, Ghebremeskel T, Seulu F, Mengistu S, Fekadu H (2003). Larval habitat diversity and ecology of anopheline larvae in Eritrea.. J Med Entomol.

[42] Weinbren MP, Masson PJ (1957). Rift Valley fever in a wild field rat (*Arvicanthis abyssinicus*): a possible natural host.. South Afr Med J.

[43] Youssef BZ, Donia HA (2002). The potential role of *Rattus rattus* in enzootic cycle of Rift Valley Fever in Egypt 2-application of reverse transcriptase polymerase chain reaction (RT-PCR) in blood samples of *Rattus rattus*.. J Egypt Public Health Assoc.

[44] Abd El-Rahim IHA, El-Hakim UA, Hussein M (1999). An epizootic of Rift Valley fever in Egypt in 1997.. Rev Sci Tech Off Int Epi.

[45] Shoemaker T, Boulianne C, Vincent MJ, Pezzanite L, Al-Qahtani MM (2002). Genetic analysis of viruses associated with emergence of Rift Valley fever in Saudi Arabia and Yemen, 2000–2001.. Emerg Infect Dis.

[46] Carroll S, Reynes J, Khristova M, Andriamandimby S (2011). Genetic evidence for Rift Valley fever outbreaks in Madagascar resulting from virus introductions from the East African mainland rather than enzootic maintenance.. J Virol.

