# Supplementary material for: An Unexpected Recurrent Transmission of Rift Valley Fever Virus in Cattle in a Temperate and Mountainous Area of Madagascar
Source: PLoS Negl Trop Dis. 2011 Dec 20;5(12):e1423. doi: 10.1371/journal.pntd.0001423 (PMC3243698; doi:10.1371/journal.pntd.0001423)
Supplement: Table S1 — Sample size, replacement practices ratio and observed serological IgM and IgG prevalence of Rift Valley fever on cattle, Anjozorobe district, Madagascar. (DOC) [file pntd.0001423.s001.doc]

**Online supporting information file**

**Table 1: Observed serological IgM and IgG prevalence of Rift Valley fever on cattle, Anjozorobe district**

| Village identification number | Villages  names | No of breeders buying animals/ No of participating breeders | No of sampled animals | No of positive samples (IgG/IgM) | IgG prevalence rate (%) |
| --- | --- | --- | --- | --- | --- |
| 1 | Fiadanana | 0/1 | 2 | 0/0 | 0 |
| 2 | Tsarafaritra | 0/1 | 4 | 0/0 | 0 |
| 3 | Anorana | 1/5 | 7 | 1/0 | 14.3 |
| 4 | Fenoarivo | 1/6 | 13 | 2/0 | 15.4 |
| 5 | Mandrizaza | 7/8 | 19 | 3/0 | 15.8 |
| 6 | Ambongabe | 4/10 | 39 | 7/0 | 17.9 |
| 7 | Ampamoha | 1/7 | 15 | 3/0 | 20.0 |
| 8 | Antseranana | 2/2 | 5 | 1/0 | 20.0 |
| 9 | Mahazoarivo | 2/4 | 20 | 4/1 | 20.0 |
| 10 | Mangarivotra | 2/3 | 5 | 1/0 | 20.0 |
| 11 | Tsarahonenana | 2/5 | 35 | 7/0 | 20.0 |
| 12 | Amboaroihazo | 6/16 | 71 | 13/0 | 20.3 |
| 13 | Ambohimadera | 2/5 | 33 | 7/0 | 21.2 |
| 14 | Antanifotsy | 8/22 | 33 | 7/1 | 21.2 |
| 15 | Ambohitraivo | 1/4 | 18 | 4/0 | 22.2 |
| 16 | Ambohibary | 0/1 | 4 | 1/0 | 25.0 |
| 17 | Ambohimandroso | 3/5 | 8 | 2/0 | 25.0 |
| 18 | Morarano kely | 1/3 | 12 | 3/0 | 25.0 |
| 19 | Tsaramasoandro | 1/1 | 4 | 1/0 | 25.0 |
| 20 | Tsaramandroso | 2/10 | 54 | 14/0 | 25.9 |
| 21 | Tsaranierana | 1/1 | 11 | 3/0 | 27.2 |
| 22 | Ambohimiaramanana | 5/16 | 64 | 18/1 | 28.1 |
| 23 | Ambongamarina | 3/10 | 30 | 9/0 | 30.0 |
| 24 | Ankerana | 4/8 | 30 | 9/1 | 30.0 |
| 25 | Morarano | 3/8 | 22 | 8/0 | 30.3 |
| 26 | Anosimanarivo | 6/10 | 29 | 9/1 | 31.0 |
| 27 | Amboanonoka | 8/18 | 57 | 19/0 | 33.3 |
| 28 | Ampanazava | 2/2 | 12 | 4/0 | 33.3 |
| 29 | Ampasika | 0/5 | 21 | 7/0 | 33.3 |
| 30 | Saharoa | 3/5 | 12 | 4/0 | 33.3 |
| 31 | Tsaramasoandro II | 1/2 | 9 | 3/0 | 33.3 |
| 32 | Ambohimahatsinjo | 1/5 | 22 | 8/0 | 36.3 |
| 33 | Andranonahoatra | 2/3 | 11 | 4/2 | 36.3 |
| 34 | Ankazabe | 1/1 | 2 | 1/0 | 36.3 |
| 35 | Miaramasoandro | 1/4 | 8 | 3/0 | 37.5 |
| 36 | Miarinarivo | 5/10 | 29 | 12/0 | 41.4 |
| 37 | Antanisoa | 2/3 | 19 | 8/0 | 42.1 |
| 38 | Ambohijanahary | 2/9 | 33 | 15/0 | 45.4 |
| 39 | Anjozorovola | 0/5 | 21 | 10/0 | 47.6 |
| 40 | Ambatolampy | 1/9 | 31 | 15/0 | 48.3 |
| 41 | Andrebakely | 0/1 | 2 | 1/0 | 50.0 |
| 42 | Ambohitsaratany | 0/1 | 5 | 3/0 | 60 |
| 43 | Sahabevary | 2/3 | 7 | 5/0 | 71.4 |

The serological survey was performed in 894 bovines, in the Anjozorobe district, Madagascar,

2009. IgG and IgM seroprevalence rates are provided regarding to the village. For each village, additional variables are the sample size, and the replacement practices ratio
